# Analytical Bias in the Measurement of Serum 25-Hydroxyvitamin D Concentrations Impairs Assessment of Vitamin D Status in Clinical and Research Settings

**DOI:** 10.1371/journal.pone.0135478

**Published:** 2015-08-12

**Authors:** Lucinda J. Black, Denise Anderson, Michael W. Clarke, Anne-Louise Ponsonby, Robyn M. Lucas, Ausimmune Investigator Group

**Affiliations:** 1 Telethon Kids Institute, The University of Western Australia, Perth, Western Australia; 2 Centre for Metabolomics, Metabolomics Australia, The University of Western Australia, Perth, Western Australia; 3 Murdoch Childrens Research Institute, The University of Melbourne, Melbourne, Australia; 4 National Centre for Epidemiology and Population Health, Research School of Population Health, The Australian National University, Canberra, Australia; University of Oxford, UNITED KINGDOM

## Abstract

Measured serum 25-hydroxyvitamin D concentrations vary depending on the type of assay used and the specific laboratory undertaking the analysis, impairing the accurate assessment of vitamin D status. We investigated differences in serum 25-hydroxyvitamin D concentrations measured at three laboratories (laboratories A and B using an assay based on liquid chromatography-tandem mass spectrometry and laboratory C using a DiaSorin Liaison assay), against a laboratory using an assay based on liquid chromatography-tandem mass spectrometry that is certified to the standard reference method developed by the National Institute of Standards and Technology and Ghent University (referred to as the ‘certified laboratory’). Separate aliquots from the same original serum sample for a subset of 50 participants from the Ausimmune Study were analysed at the four laboratories. Bland-Altman plots were used to visually check agreement between each laboratory against the certified laboratory. Compared with the certified laboratory, serum 25-hydroxyvitamin D concentrations were on average 12.4 nmol/L higher at laboratory A (95% limits of agreement: -17.8,42.6); 12.8 nmol/L higher at laboratory B (95% limits of agreement: 0.8,24.8); and 10.6 nmol/L lower at laboratory C (95% limits of agreement: -48.4,27.1). The prevalence of vitamin D deficiency (defined here as 25-hydroxyvitamin D <50 nmol/L) was 24%, 16%, 12% and 41% at the certified laboratory, and laboratories A, B, and C, respectively. Our results demonstrate considerable differences in the measurement of 25-hydroxyvitamin D concentrations compared with a certified laboratory, even between laboratories using assays based on liquid chromatography-tandem mass spectrometry, which is often considered the gold-standard assay. To ensure accurate and reliable measurement of serum 25-hydroxyvitamin D concentrations, all laboratories should use an accuracy-based quality assurance system and, ideally, comply with international standardisation efforts.

## Introduction

Vitamin D status is assessed by measuring serum concentrations of total 25-hydroxyvitamin D (25(OH)D), which is the sum of two metabolites, 25(OH)D_3_ and 25(OH)D_2_. Vitamin D_3_ is produced from 7-dehydrocholesterol in the skin on exposure to sunlight and is hydroxylated to 25(OH)D_3_ in the liver [1]. Some foods (namely fish, meat, eggs and dairy) and supplements contain vitamin D_3_, while mushrooms and other supplements contain vitamin D_2_, which is metabolised to 25(OH)D_2_. An epimer of 25(OH)D_3_, 3-epi-25(OH)D_3_, has been identified and its biological activity is unknown [2]; however, the general consensus is to exclude 3-epi-25(OH)D_3_ when assessing vitamin D status [3].

Given the current interest in vitamin D status and its purported relationship with a wide range of health outcomes, the accurate and reliable assessment of serum 25(OH)D concentrations, in both research and clinical settings, is imperative. A high prevalence of vitamin D deficiency has been reported in populations worldwide based on measurement of serum 25(OH)D concentrations [4–8]. In epidemiological studies, low serum 25(OH)D concentrations have been proposed as a risk factor for a variety of skeletal and non-skeletal conditions, although the evidence is largely inconclusive [9]. In clinical practice, deficient or insufficient vitamin D status may signify a need for lifestyle and/or dietary changes, including supplementation.

Serum 25(OH)D concentrations are measurable using several analytical techniques, including competitive protein binding assay [10,11], high-performance liquid chromatography [12,13], radioimmunoassay [14], enzyme immunoassay [15] and the more recent assays based on liquid chromatography-tandem mass spectrometry (LC-MS/MS) [16]. LC-MS/MS-based assays are the most sensitive and specific for measuring serum 25(OH)D concentrations, but the high equipment costs have limited widespread clinical use. Although clinical laboratories largely rely on automated immunoassays, their accuracy and precision varies widely [17], leading to a general acceptance that an LC-MS/MS-based assay is the gold-standard [18]. However, using LC-MS/MS does not guarantee accurate and reliable results; rather, the validity of measurements derived from any assay is dependent on the specific analytical method, quality control, instrument maintenance and equipment calibration.

An international effort to standardise the measurement of 25(OH)D and its metabolites is currently being led by the Vitamin D Standardization Program (VDSP), which was established in November 2010 by the National Institutes of Health Office of Dietary Supplements, the Centers for Disease Control and Prevention (CDC), the National Institute of Standards and Technology (NIST) and Ghent University [19]. The aim of standardisation is to bring laboratories into alignment with the “true” value as measured by the reference measurement procedure (RMP) developed by NIST and Ghent University. In this study, we investigate the differences in serum 25(OH)D concentrations measured at three laboratories (two using LC-MS/MS-based assays and one using DiaSorin Liaison) compared with a laboratory using an LC-MS/MS-based assay that is certified to the RMP. We highlight the scientific and clinical implications of relying on serum 25(OH)D concentrations measured by uncertified laboratories. We also discuss some of the difficulties inherent in measuring serum 25(OH)D concentrations, including quality control and equipment calibration.

## Materials and Methods

### Study participants

The Australian Multicentre Study of Environment and Immune Function (the Ausimmune Study) is a matched case-control study examining environmental risk factors for the onset of central nervous system demyelinating disease [20]. In brief, 840 participants aged 18–59 years (22% male, 78% female) were recruited from four geographical regions in Australia (Brisbane, Newcastle, Geelong and Tasmania). Cases were persons with an incident first demyelinating event and controls were matched on sex, age (within two years) and region of residence. Venous blood samples (15 mL) were collected from 813 participants (both cases and controls) between January 2004 and July 2007 and stored in 1 mL aliquots at -80°C.

### Ethics Statement

Ethics approval was obtained from the nine Human Research Ethics Committees of the participating institutions, namely: Barwon Health Research and Ethics Advisory Committee (ref 03/46); Ballarat Health Services and St John of God Health Services Committee; Royal Brisbane & Women’s Hospital & Health Service District Office of the Human Research Ethics Committee (ref 2003/093); The University of Queensland Medical Research Ethics Committee (ref 2003000253); The Princess Alexandra Hospital Human Research Ethics Committee (ref 2004/059); The Queensland institute of Medical Research Human Research Ethics Committee (ref H0511-061 (P950)); Hunter Area Research Ethics Committee (ref 03/06/11/3.07); Southern Tasmania Heath and Medical Research Ethics Committee (ref H7436); and The Australian National University Human Research Ethics Committee (ref 2002/111). All participants gave written informed consent for the use of their data and for their blood samples to be tested for "vitamin D measurements". In addition, all participant information was anonymized and de-identified prior to analysis.

### Sample selection

Serum 25(OH)D concentrations in 812 of the 813 samples were originally analysed at laboratory C using DiaSorin Liasion in September 2007 (one sample was missed). All 813 samples were re-analysed at laboratory A using an LC-MS/MS-based assay, between October 2008 and May 2009. We previously reported the agreement in 25(OH)D concentrations measured by the two different assays [17]. A total of 50 samples were then chosen uniformly across quintiles of the serum 25(OH)D concentrations reported by laboratory A, with ten samples chosen from each quintile. For these 50 samples, serum 25(OH)D concentrations from separate aliquots of the same original serum sample were analysed at laboratory B using an LC-MS/MS-based assay, and at the CDC (also an LC-MS/MS-based assay), which is certified to the RMP developed by NIST and Ghent University (referred to from now on as the ‘certified laboratory’). Results for only 49 of these samples were available from laboratory C, due to the initial missing sample.

### Analysis of vitamin D metabolites

The certified laboratory, along with laboratories A and B, used an isotope-dilution LC-MS/MS method to quantify 25(OH)D [16]. Laboratory C quantified 25(OH)D by chemiluminescence using a two-step incubation process with human serum calibrators (DiaSorin Liaison kit insert). The analytical characteristics (inter-assay Coefficients of Variation, CV; analytical Limit of Detection, LOD) of the laboratories using LC-MS/MS-based assays are shown in Table 1.

**Table 1:** Analytical characteristics of the laboratories using assays based on liquid chromatography-tandem mass spectrometry.

Characteristic	Certified laboratory	Laboratory A	Laboratory B
**Inter-assay CV**			
** 25(OH)D**_**3**_	1.9% at 29.7 nmol/L	5.5% at 25.0 nmol/L	12.7% at 25.0 nmol/L
	4.5% at 52.6 nmol/L	3.9% at 160.9 nmol/L	13.7% at 53.7 nmol/L
	2.9% at 92.2 nmol/L	6.4% at 467.1 nmol/L	8.8% at 102.7 nmol/L
			6.4% at 196.6 nmol/L
** 25(OH)D**_**2**_	6.2% at 5.0 nmol/L	13.9% at 9.7 nmol/L	NR
	5.4% at 10.8 nmol/L	13.5% at 31.1 nmol/L	NR
	6.6% at 21.6 nmol/L	8.5% at 80.7 nmol/L	NR
** 3-epi-25(OH)D**_**3**_	8.1% at 3.2 nmol/L	NR	NR
	8.9% at 5.6 nmol/L	NR	NR
	10.2% at 15.8 nmol/L	NR	NR
**LOD**			
** 25(OH)D**_**3**_	2.2 nmol/L	5.0 nmol/L	3.0 nmol/L
** 25(OH)D**_**2**_	2.1 nmol/L	2.7 nmol/L	5.0 nmol/L
** 3-epi-25(OH)D**_**3**_	1.6 nmol/L	NR	NR

CV, Coefficients of Variation; LOD, analytical Limit of Detection; 25(OH)D_3_, 25-hydroxyvitamin D_3_; 25(OH)D_2_, 25-hydroxyvitamin D_2_; 3-epi-25(OH)D_3_, c3 epimer; NR, not reported.

Determinations of serum vitamin D metabolites (25(OH)D_2_, 25(OH)D_3_ and 3-epi-25(OH)D_3_) were performed from August to October 2013 at the certified laboratory using the Vit D2 D3 NBB 4027 LC-MS/MS method. All patient serum samples were analysed in singlicate. Minimal bias of the method was established by periodic measurement of NIST SRM 972a, and the laboratory successfully passed the performance criterion of ±5% mean bias to the RMP and an overall imprecision of <10% over the concentration range of 22–275 nmol/L for total 25(OH)D. This laboratory also successfully participates in the NIST Vitamin D Metabolites Quality Assurance Program, the UK Vitamin D External Quality Assessment Scheme (DEQAS), and several College of American Pathologists programs. Quality control for these determinations was established by performing duplicate analysis of three in-house serum QC pools, assayed at the beginning and the end of the run. The intra-assay CVs were 5.0% for 25(OH)D_3_, 9.7% for 25(OH)D_2_ and 8.4% for 3-epi-25(OH)D_3_. Compared with the SRM 972a, the bias was -1.4% for 25(OH)D_3_, -4.4% for 25(OH)D_2_ and -3.0% for 3-epi-25(OH)D_3_. Compared with the SRM 972a, the inter-assay CVs were 4.6% for 25(OH)D_3_, 10.8% for 25(OH)D_2_ and 2.8% for 3-epi-25(OH)D_3_. Note that the majority of samples were below the LOD for 25(OH)D_2_.

At Laboratory A, the method for the quantification of vitamin D metabolites (25(OH)D_2_ and 25(OH)D_3_) in serum/plasma used hexa-deuterated 25(OH)D_3_ as an internal standard on either the API 4000 QTRAP system or the Agilent 6410 QQQ LC-MS/MS system. All patient serum samples were analysed in singlicate. Serum Calibration Standards containing nominal amounts of 25(OH)D_2_/25(OH)D_3_ from CHROMSYSTEMS were used to generate calibration curves. Tri-level Quality Control samples containing 25(OH)D_2_/25(OH)D_3_ designated as Level Low, Level 1 and Level 2 were purchased from UTAK Laboratories (PM Separations). The intra-assay CVs for 25(OH)D_2_ were 2.7% at 8.5 nmol/L, 8.1% at 31.57 nmol/L and 0.5% at 94.8 nmol/L.

Determinations of serum 25(OH)D concentrations were performed by laboratory B (LC-MS/MS) in November 2012. All patient serum samples were analysed in singlicate. Laboratory B uses an external quality control provided by DEQAS and precision was calculated based on the quality controls, which were assayed once per batch, at the beginning and the end. No intra-assay CVs were available.

Laboratory C (DiaSorin Liaison) analysed serum 25(OH)D concentrations in singlicate in September 2007. The CVs at laboratory C varied between 9 and 12% across the range of 25(OH)D concentrations, between 13 and 143 nmol/L.

### Statistical analysis

We compared only the results for 25(OH)D_3_. Results for 25(OH)D_2_ were excluded from the comparisons because the concentrations of this metabolite were below the LOD in 44 of the 50 samples at the certified laboratory, 46 of the 50 samples at laboratory A, and in all 50 samples at laboratory B. Although the DiaSorin Liaison used at laboratory C has the capacity to detect both 25(OH)D_2_ and 25(OH)D_3_, it does not separately quantify them. However, detection of very low levels of 25(OH)D_2_ by this assay would be unlikely. Therefore, we compared 25(OH)D_3_ concentrations from the certified laboratory with 25(OH)D concentrations reported by laboratory C.

The certified laboratory separated 3-epi-25(OH)D_3_ from 25(OH)D_3_, while laboratories A and B detected 3-epi-25(OH)D_3_ but did not quantify it separately to 25(OH)D_3_. Therefore, in order to ensure a fair comparison, we added the 3-epi-25(OH)D_3_ and 25(OH)D_3_ results from the certified laboratory before comparing with the results from laboratories A and B. In contrast, the assay used at laboratory C does not detect 3-epi-25(OH)D_3_; therefore, we excluded the 3-epi-25(OH)D_3_ results reported by the certified laboratory when comparing with laboratory C.

All statistical analyses were undertaken using R version 3.0.1. [21]. Bland-Altman plots [22] were used to check agreement between each pairwise comparison of assays with plots generated using the plotDifference() function available in the method comparison regression (mcr) package [23]. Limits of agreement were calculated as the mean difference plus or minus twice the standard deviation of the differences. Weighted Deming regression models were fitted to assess systematic bias between assays using the mcreg() function available in the mcr package. For these models, the ratio between the squared measurement errors of both assays was assumed to be 1 and the confidence interval for model parameter estimates was calculated using the jackknife method [24]. For each laboratory, we determined the number of individuals within specific categories of 25(OH)D concentrations: <13, 13–29, 30–49, 50–74 and ≥ 75 nmol/L, with deficiency defined as < 50 nmol/L [4].

## Results

**Fig 1:**
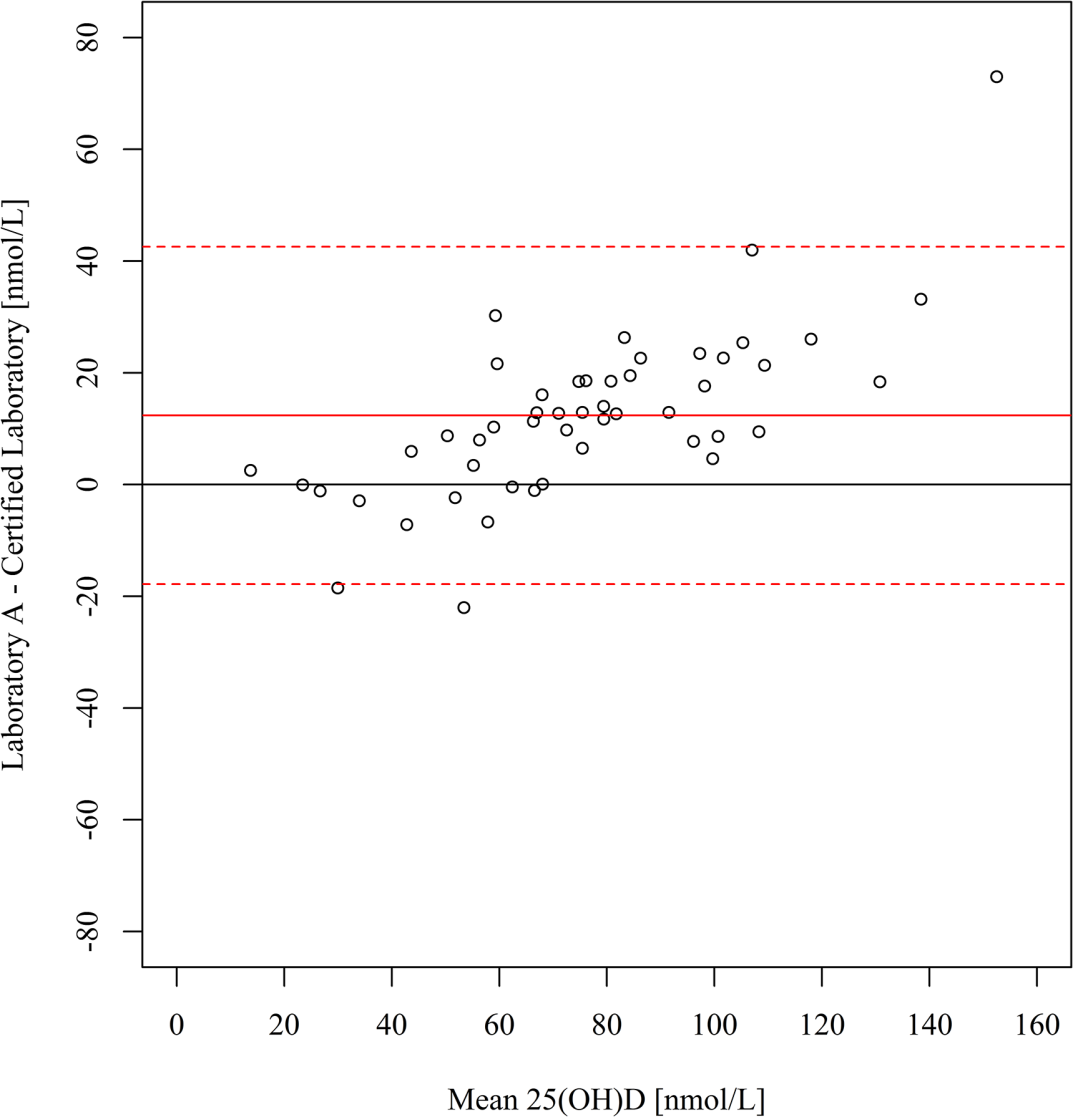
Bland-Altman plot of serum 25(OH)D concentrations at laboratory A (LC-MS/MS) *v* certified laboratory (LC-MS/MS). The solid lines indicate the mean bias and the dotted lines indicate the 95% limits of agreement. 25(OH)D, 25-hydroxyvitamin D.

**Fig 2:**
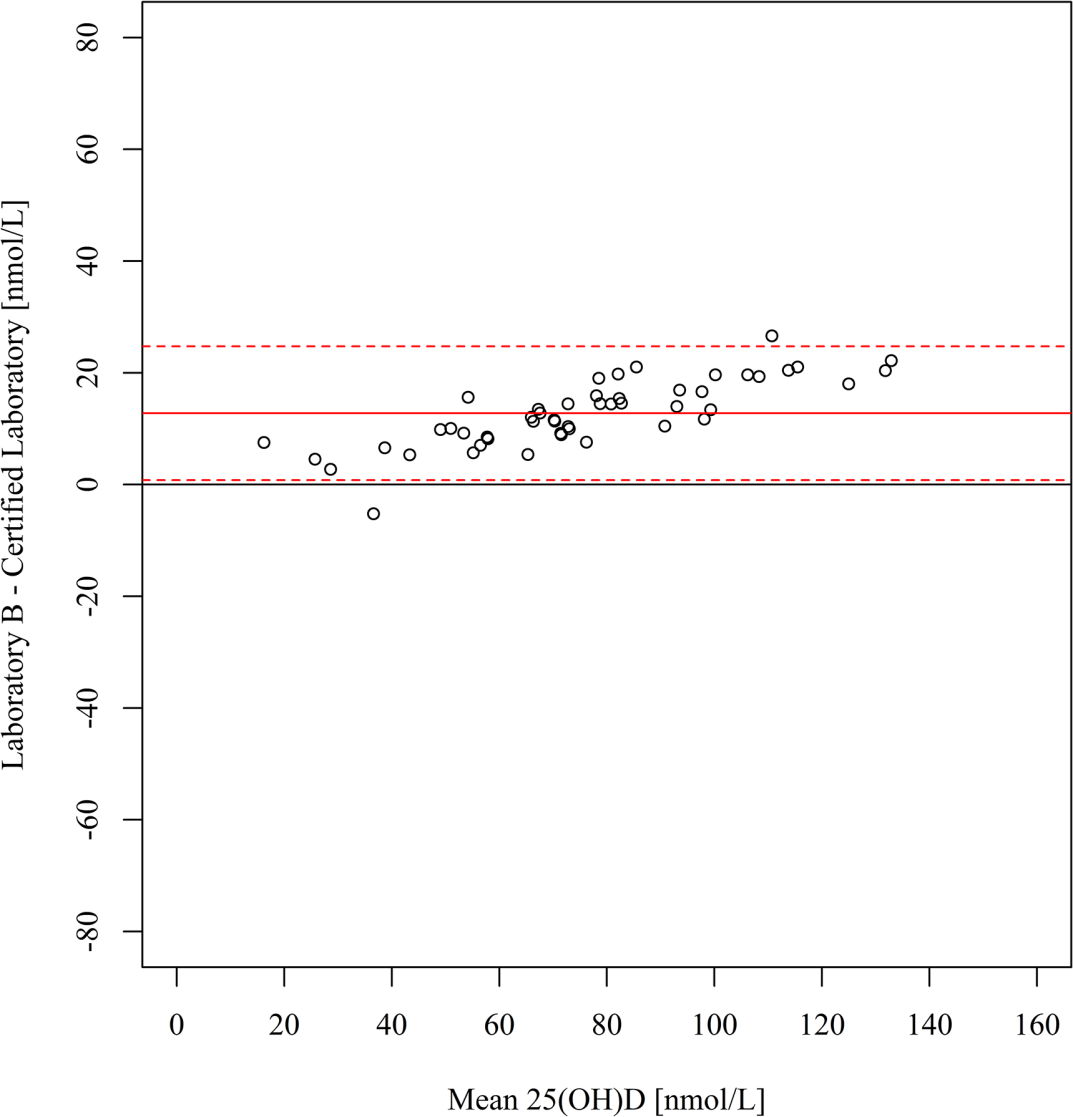
Bland-Altman plot of serum 25(OH)D concentrations at laboratory B (LC-MS/MS) *v* certified laboratory (LC-MS/MS). The solid lines indicate the mean bias and the dotted lines indicate the 95% limits of agreement. 25(OH)D, 25-hydroxyvitamin D.

**Fig 3:**
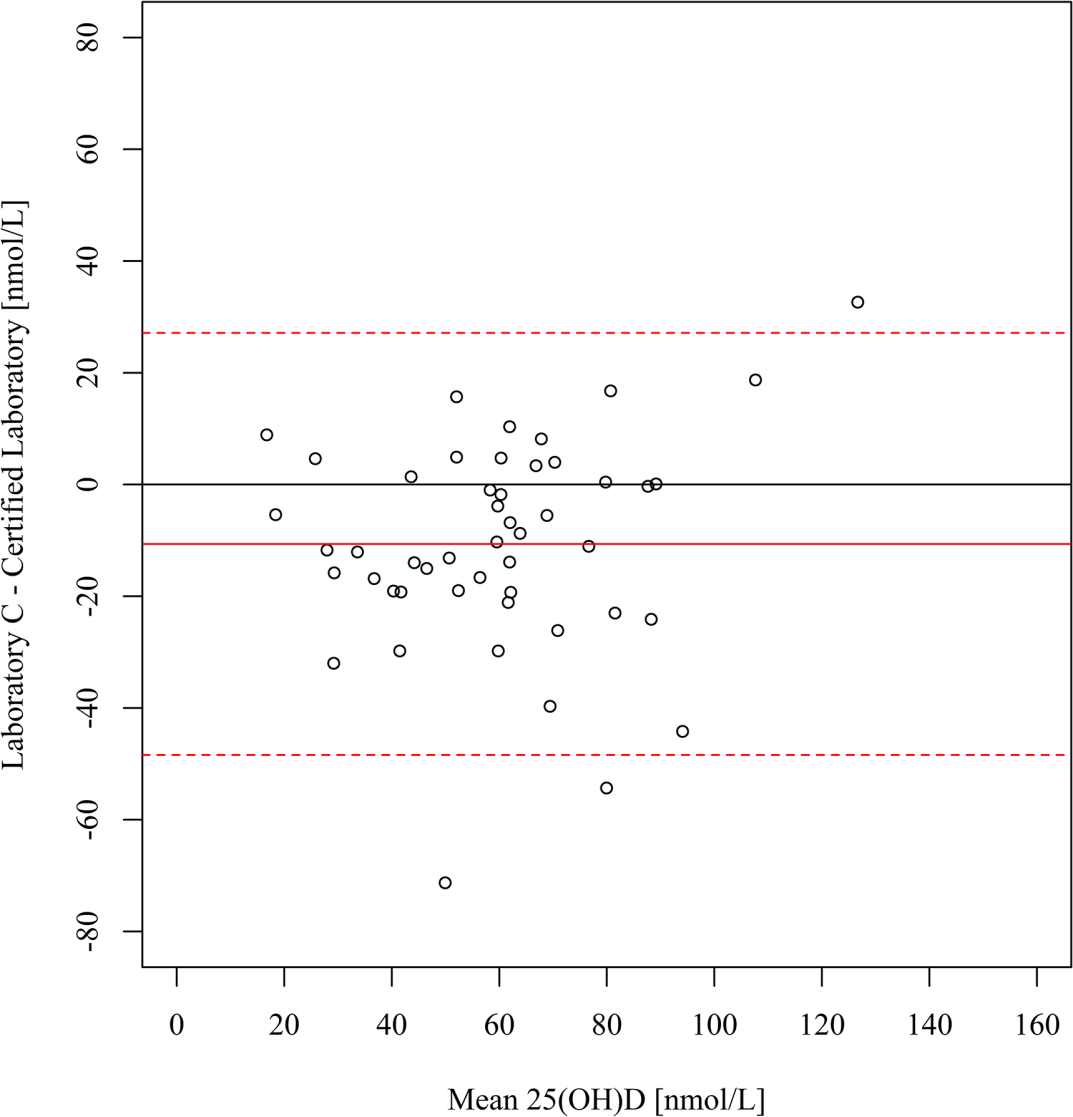
Bland-Altman plot of serum 25(OH)D concentrations at laboratory C (DiaSorin Liaison) *v* certified laboratory (LC-MS/MS). The solid lines indicate the mean bias and the dotted lines indicate the 95% limits of agreement. 25(OH)D, 25-hydroxyvitamin D

**Fig 4:**
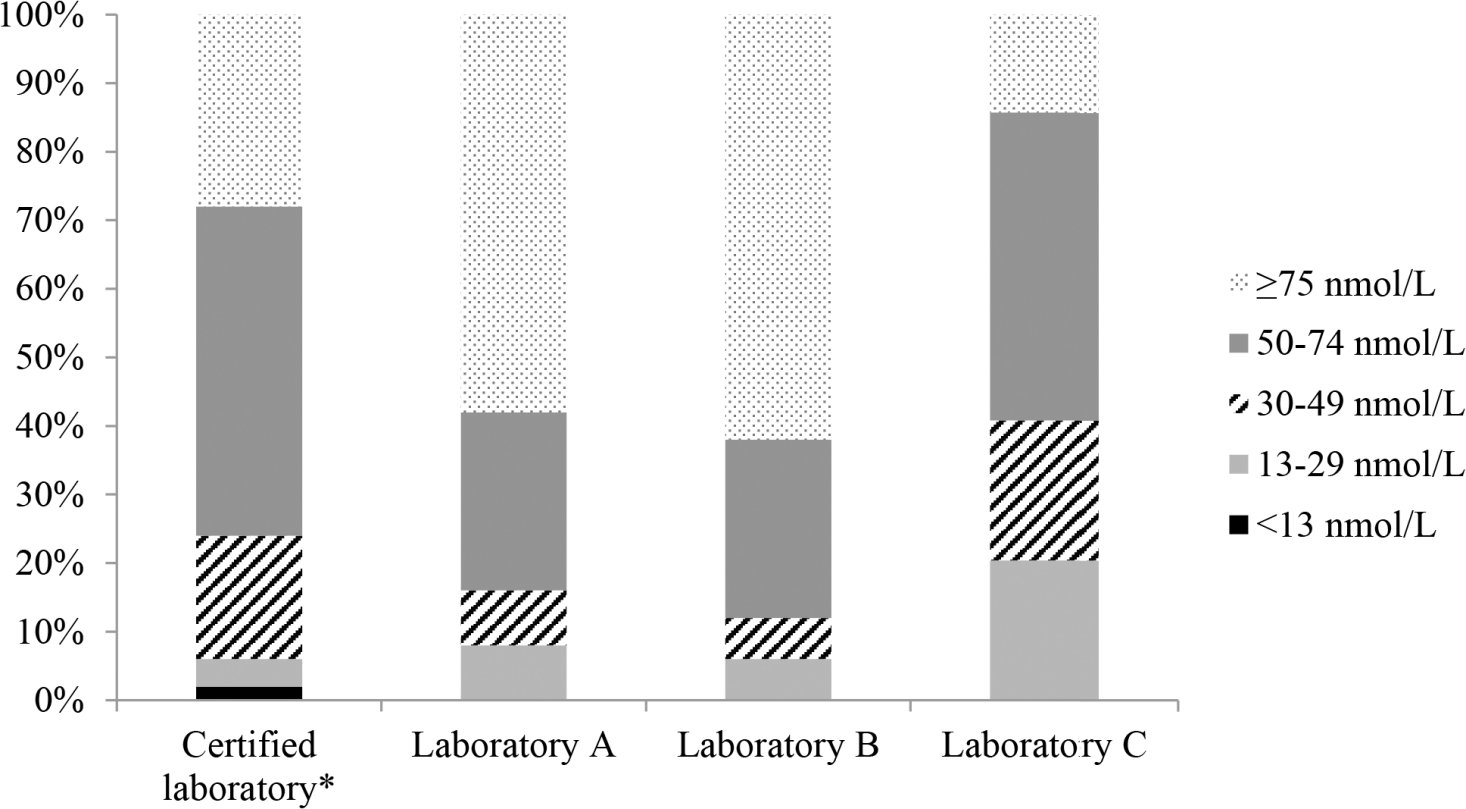
Estimates of vitamin D deficiency based on results reported by the certified laboratory and laboratories A-C. Total deficiency <50 nmol/L. *Given that the general consensus is to exclude 3-epi-25(OH)D_3_ when assessing vitamin D status, values for 3-epi-25(OH)D_3_ from the certified laboratory were excluded.

Table 2 shows the range of 25(OH)D concentrations reported by each laboratory, along with descriptive statistics. Compared with the certified laboratory, the serum 25(OH)D concentrations were on average 12.4 nmol/L higher at laboratory A (95% limits of agreement: -17.8, 42.6) (Fig 1) and 12.8 nmol/L higher at laboratory B (95% limits of agreement: 0.8, 24.8) (Fig 2). The magnitude of these differences increased at higher 25(OH)D concentrations, as confirmed by the corresponding weighted Deming regression analyses (S1 Fig). Compared with the certified laboratory, the serum 25(OH)D concentrations were on average 10.6 nmol/L lower at laboratory C (95% limits of agreement: -48.4, 27.1) (Fig 3). The prevalence of deficiency (<50 nmol/L) based on results from the certified laboratory and laboratories A, B and C, was 24%, 16%, 12% and 41%, respectively (Fig 4).

**Table 2:** Descriptive statistics for vitamin D metabolites reported by the certified laboratory and laboratories A-C.

Laboratory	Metabolite	*n*	Range (nmol/L)	Mean (nmol/L)	SD (nmol/L)	Median (nmol/L)	IQR (nmol/L)
**Certified laboratory** ^a^	25(OH)D_3_	*50*	12.3–116.2	65.5	22.7	64.7	31.8
	3-epi-25(OH)D_3_	*50*	0.2–11.5	4.1	2.4	3.5	3.2
**Laboratory A** ^a^	25(OH)D_3_	*50*	15.0–189.0	82.0	35.0	80.3	43.3
**Laboratory B** ^a^	25(OH)D_3_	*50*	20.0–144.0	82.4	29.1	79.0	40.5
**Laboratory C** ^b^	25(OH)D	*49*	13.2–143.0	54.4	25.6	54.5	34.6

25(OH)D_3_, 25-hydroxyvitamin D_3_; 3-epi-25(OH)D_3_, c3 epimer; 25(OH)D, 25-hydroxyvitamin D

^a^Liquid chromatography-tandem mass spectrometry

^b^DiaSorin Liaison

## Discussion

To our knowledge, this is the first study comparing 25(OH)D concentrations from laboratories using LC-MS/MS-based assays with a laboratory certified to the RMP developed by NIST and Ghent University. Our results demonstrate substantial positive measurement bias between two uncertified laboratories using LC-MS/MS-based methods and the certified laboratory. In accordance with other published literature [17,25], we found a substantial negative measurement bias for the DiaSorin Liaison compared with the LC-MS/MS-based assays, although the difference was not as great as we have previously reported [17].

It is important to be aware that, although LC-MS/MS-based assays are widely considered the gold standard assay for the measurement of 25(OH)D [18], the performance of the assay is highly dependent on the performance of the specific laboratory. Variable results may be attributed to differences in calibration and/or sample preparation [26,27]. When deciding on which assay to use for the measurement of serum 25(OH)D concentrations, it is imperative to check the performance characteristics of the assay in the specific laboratory. For all methods, incomplete release of the metabolite from the vitamin D-binding protein will lead to an under-recovery of the metabolite measured. There can also be differences between the sample and the choice of calibrator and how that is made (e.g. spiked serum *v* spiked human serum albumin *v* methanol). This leads to differences in recovery and, in the case of LC-MS/MS, ion suppression effects caused by the sample that might be slightly different to the calibrator. Coupled to this is the choice of calibrator and whether this is standardised to the NIST vitamin D standard, SRM 972a. Accuracy-based quality assurance schemes such as DEQAS and the CDC standardisation program provide metrics for the accuracy of the assay against the RMP developed by NIST and Ghent University.

Some LC-MS/MS-based methods are sensitive enough to detect 3-epi-25(OH)D_3,_ but laboratories may or may not quantify it separately from 25(OH)D_3,_ as this involves chromatographic separation of the two molecules [25]. Recent data from a nationally representative sample of adults participating in the Irish National Adult Nutrition Survey (n = 1132) showed that the vast majority of participants had quantifiable serum 3-epi-25(OH)D_3_ [3]. That study showed a significant positive correlation between serum 3-epi-25(OH)D_3_ and serum 25(OH)D_3_ in those who had serum 3-epi-25(OH)D_3_ higher than the limit of quantification (*r* = 0.78; p < 0.001; *n* = 1082); however, there was a large variability in serum 3-epi-25(OH)D_3_ at any one concentration of serum 25(OH)D_3,_ particularly as serum 25(OH)D_3_ concentration increased. Although the physiologic functions of 3-epi-25(OH)D_3_ are unclear [2], it is currently thought that this metabolite should not be included in estimates of vitamin D status [3]. The inclusion of 3-epi-25(OH)D_3_ in the assessment of vitamin D status serves to inflate the estimate of 25(OH)D_3_ and may mask low values of clinical and public health importance. The prevalence of vitamin D deficiency in populations may be underestimated if the assay used to quantify 25(OH)D does not separate 3-epi-25(OH)D_3_. In the present study, the only laboratory to separately quantify 3-epi-25(OH)D_3_ was the certified laboratory. However, we accounted for the presence of the epimer in our comparisons, ensuring that any differences between assays were not due to 3-epi-25(OH)D_3_.

Immunoassays have the advantage of automation and have substantially higher throughput than the LC-MS/MS methods. They work through an antibody binding to the carbon skeleton of 25(OH)D, often C1-C22; the antibody binding is then detected by measurement of a marker. The binding is not specific for 25(OH)D_3_ and, as noted, also picks up 25(OH)D_2_. Equally, these antibodies also bind to other vitamin D metabolites that have the same C1-C22 conformation, such as the catabolic product, 24,25(OH)_2_D, which is commonly present in nanomolar amounts. A recent investigation demonstrated that serum 24,25(OH)_2_D is indeed an interferent for some immunoassays [28]. However, if purposefully measured, this metabolite may have clinical relevance by serving as a marker of vitamin D deficiency and catabolism in healthy individuals, and by helping to explain the observed differences in response to vitamin D supplementation [29].

There are a number of potential problems with immunoassays that may result in both inaccuracy and imprecision, including how the method is standardised, instrument maintenance and water quality. Although immunoassays generally under-recover 25(OH)D_2_, this is unlikely to cause a problem in countries such as Australia, where vitamin D supplements are almost exclusively in the form of vitamin D_3_. In contrast, in the United States and other countries that use vitamin D_2_ in supplements or fortified foods, the accurate quantification of 25(OH)D_2_ may be important, particularly for monitoring treatment of vitamin D deficiency with supplementation. The data in the current study are based on a prior version of the commonly-used DiaSorin Liaison. In recent years, the assay has been reformulated and is now accredited by the VDSP as certified to the NIST standard, which is a positive step towards standardisation of commercially-used assays.

Reliability (precision) and accuracy are of particular concern in epidemiological studies where poor precision leads to non-differential misclassification bias and may cause null findings. Furthermore, research on the risks of vitamin D deficiency for human health outcomes often involves combining the results of many different studies, where accuracy may be a particular issue. For example, a latitudinal gradient in 25(OH)D concentrations [30] lends support to vitamin D deficiency as a risk factor for diseases that themselves show a latitude gradient [31]. In addition, meta-analyses examine the consistency of results across studies and estimate a summary measure of effect. What is clear from our analyses is that it is not sufficient to take account just of assay type; this will not take account of the variability between assays, as there is considerably variation in results even within the same assay at different laboratories and possibly at different times. Researchers should be aware of the limitations of vitamin D assays and the implications for the analysis of results, particularly with multiple sampling over time, and when comparing results from different assays, different laboratories, or where different batches of reagent kits are used.

Importantly, population estimates of vitamin D deficiency may be unreliable if they are based on a non-certified assay. Assays that read low are likely to overestimate the prevalence of vitamin D deficiency in population groups, potentially leading to unnecessary implementation of public health measures, such as food fortification. In contrast, assays that read high may mask a public health problem due to the underestimation of vitamin D deficiency. Subject to the availability of banked sera, it is possible to remeasure selected samples from health surveys using a certified assay and create regression equations that allow the initial results to be adjusted in value to accord with results from the certified assay. The VDSP has developed a protocol to support this harmonisation [19]; this technique has recently been evaluated using data from the Irish National Adult Nutrition Survey [32]. Regression equations were created using the initial results and those from re-measurement of 99 stored serum samples at a certified laboratory. This allowed prediction of the ‘real’ 25(OH)D level for all 1118 samples. All of these original samples were then reanalysed at the certified laboratory. The predicted and measured (at the certified laboratory) results showed close agreement and the authors concluded that the VDSP harmonisation protocols hold major potential for the standardisation of existing serum 25(OH)D data from nutrition and health surveys.

In Australia, growing interest in the health risks of vitamin D deficiency has led to an exponential increase in the measurement of serum 25(OH)D concentrations to assess vitamin D status [33]. The number of claims to Medicare (the national medical care funder) for vitamin D testing in Australia has increased each year over the past ten years, from fewer than 120,000 claims in 2003/2004 to over 4 million claims in 2012/13. This is a 3587% increase in vitamin D testing services [34]. The majority of tests are being requested by general practitioners and other medical practitioners for the purposes of screening, rather than follow-up monitoring. Over the same period, a similar increase (3450%) was seen in benefits paid by Medicare, which rose from less than $5 million in 2003/04 to over $150 million in 2012/2013 [34]. In response, the Australian government recently changed the eligibility for a Medicare rebate for routine vitamin D testing. Rebates for quantification of 25(OH)D in serum are now available only for patients at high risk of vitamin D deficiency e.g. the patient has signs or symptoms of rickets or osteomalacia, has deeply pigmented skin or severe lack of sun exposure [34]. However, given the current scientific and media interest in vitamin D, doctors may continue to request vitamin D tests for a large proportion of patients they consider at risk of vitamin D deficiency, despite these new eligibility criteria.

The clinical implication of using a method that is not certified to the RMP developed by NIST and Ghent University, where accuracy cannot be assured, is the misdiagnosis of vitamin D deficiency. If an assay reads low, there is the potential to supplement patients who are not actually vitamin D deficient; if an assay reads high, some patients who need supplementation may not be treated. Currently, the vast majority of commercial laboratories rely on automated immunoassays, many of which are known to read low compared with LC-MS/MS [17, 25]. This raises the concern that some patients may be receiving treatment for vitamin D deficiency unnecessarily. Clinicians should be aware of these issues since they generally have only a single vitamin D test on which to base their management decisions. Such decisions should be made by taking the vitamin D result within the context of the patient’s history of time outdoors, the location, skin type and any co-morbidities.

## Conclusions

Our research demonstrates substantial differences in reported serum 25(OH)D concentrations compared with a certified laboratory, even amongst laboratories using an LC-MS/MS assay. Both researchers and clinicians should be aware of the issues when relying on measurements of serum 25(OH)D concentrations from a non-certified laboratory. In order to ensure accurate and reliable measurement of serum 25(OH)D concentrations, all laboratories should monitor their performance with an accuracy-based quality assurance system, with regular checks, and should, ideally, comply with international standardisation efforts.

## Supporting Information

S1 Fig Deming regressions of: a) Serum 25 (OH)D concentrations at laboratory A (LC-MS/MS) *v* certified laboratory (LC-MS/MS). b) Serum 25 (OH)D concentrations at laboratory B (LC-MS/MS) *v* certified laboratory (LC-MS/MS). c) Serum 25 (OH)D concentrations at laboratory C (DiaSorin Liaison) *v* certified laboratory (LC-MS/MS). Dotted lines show perfect agreement between assays (intercept of 0 and slope of 1). 25(OH)D, 25-hydroxyvitamin D; LC-MS/MS, liquid chromatography-tandem mass spectrometry
(DOCX)
