# Supplementary figures and images for: Analytical Bias in the Measurement of Serum 25-Hydroxyvitamin D Concentrations Impairs Assessment of Vitamin D Status in Clinical and Research Settings
Source: PLoS One. 2015 Aug 12;10(8):e0135478. doi: 10.1371/journal.pone.0135478 (PMC4534132; doi:10.1371/journal.pone.0135478)

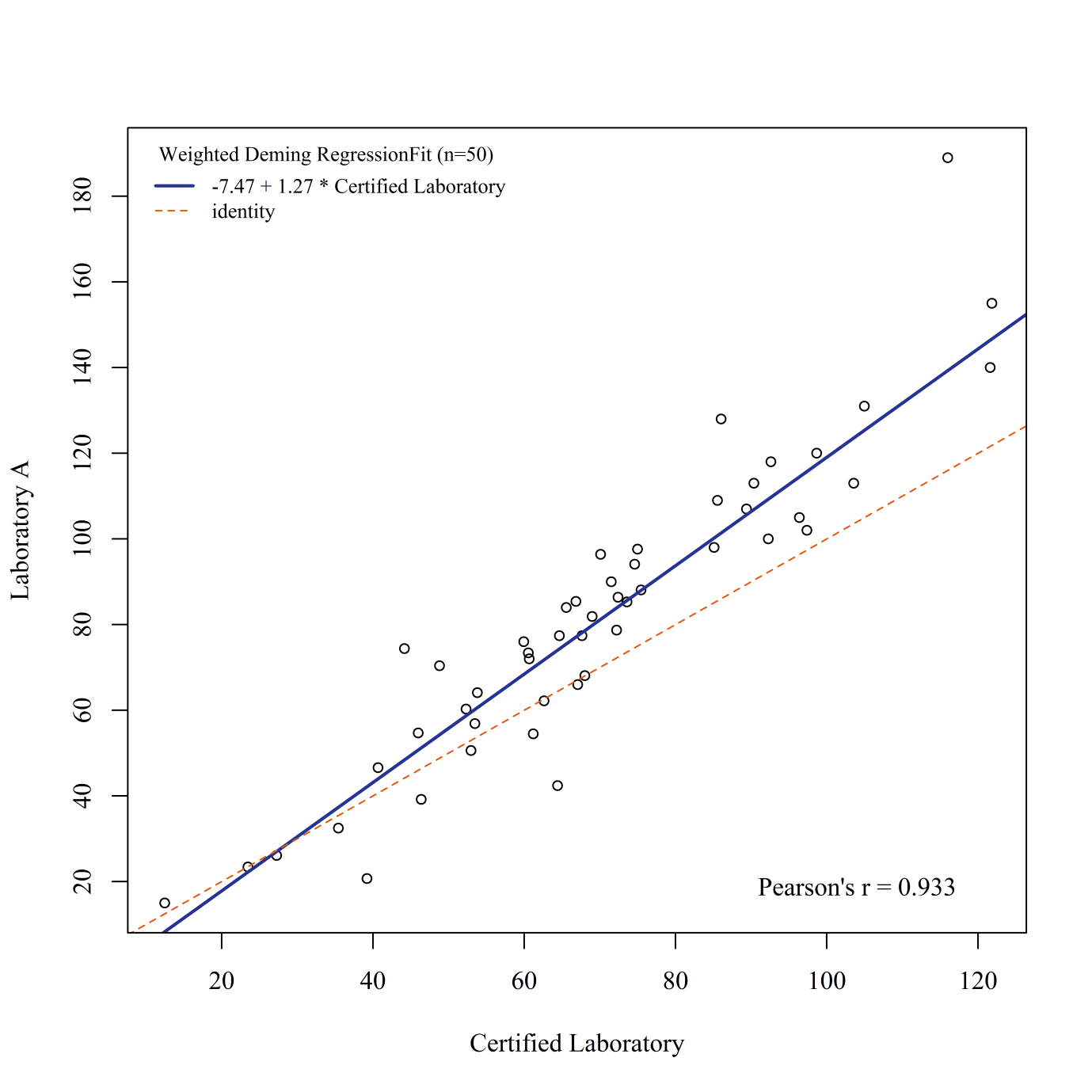
a)


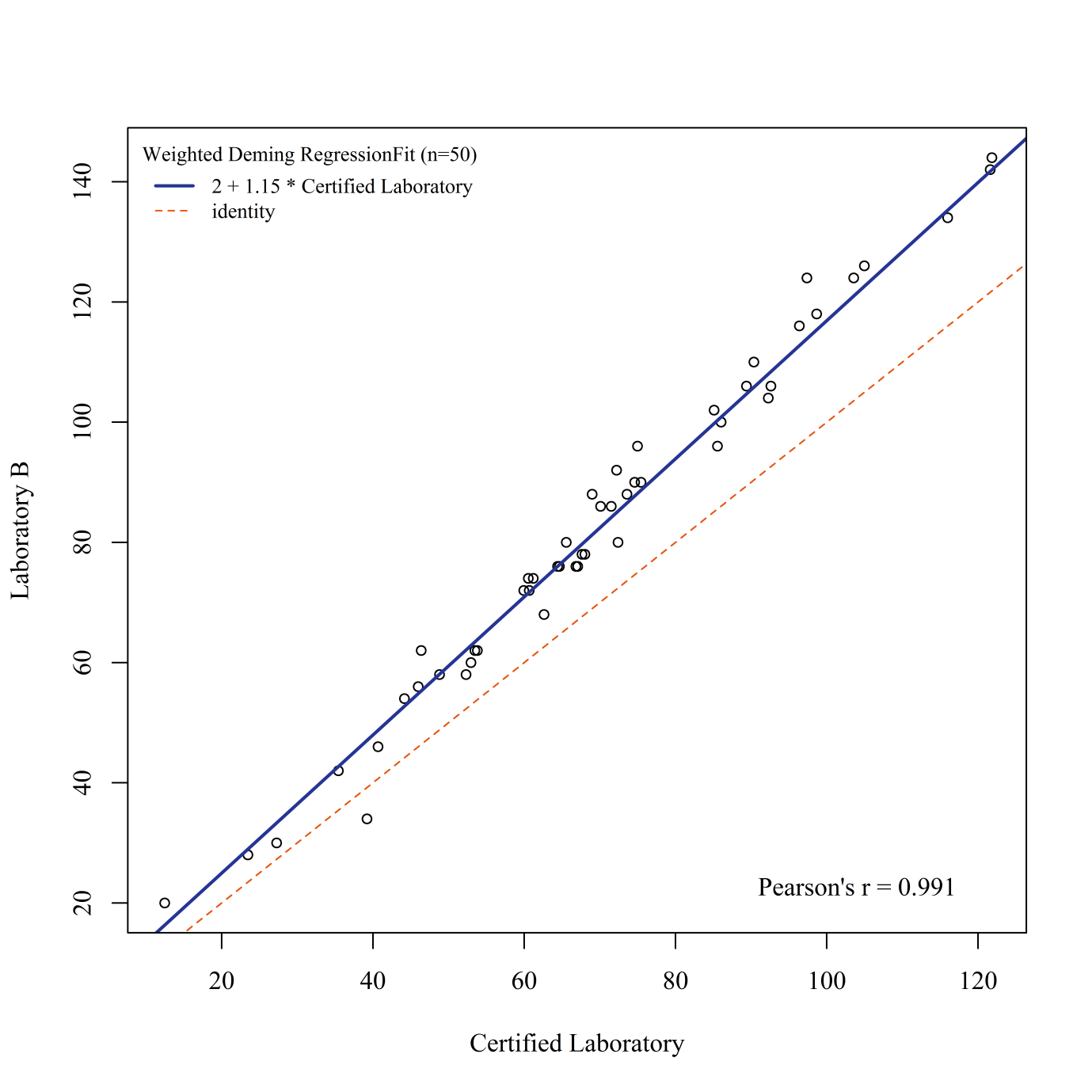


b)


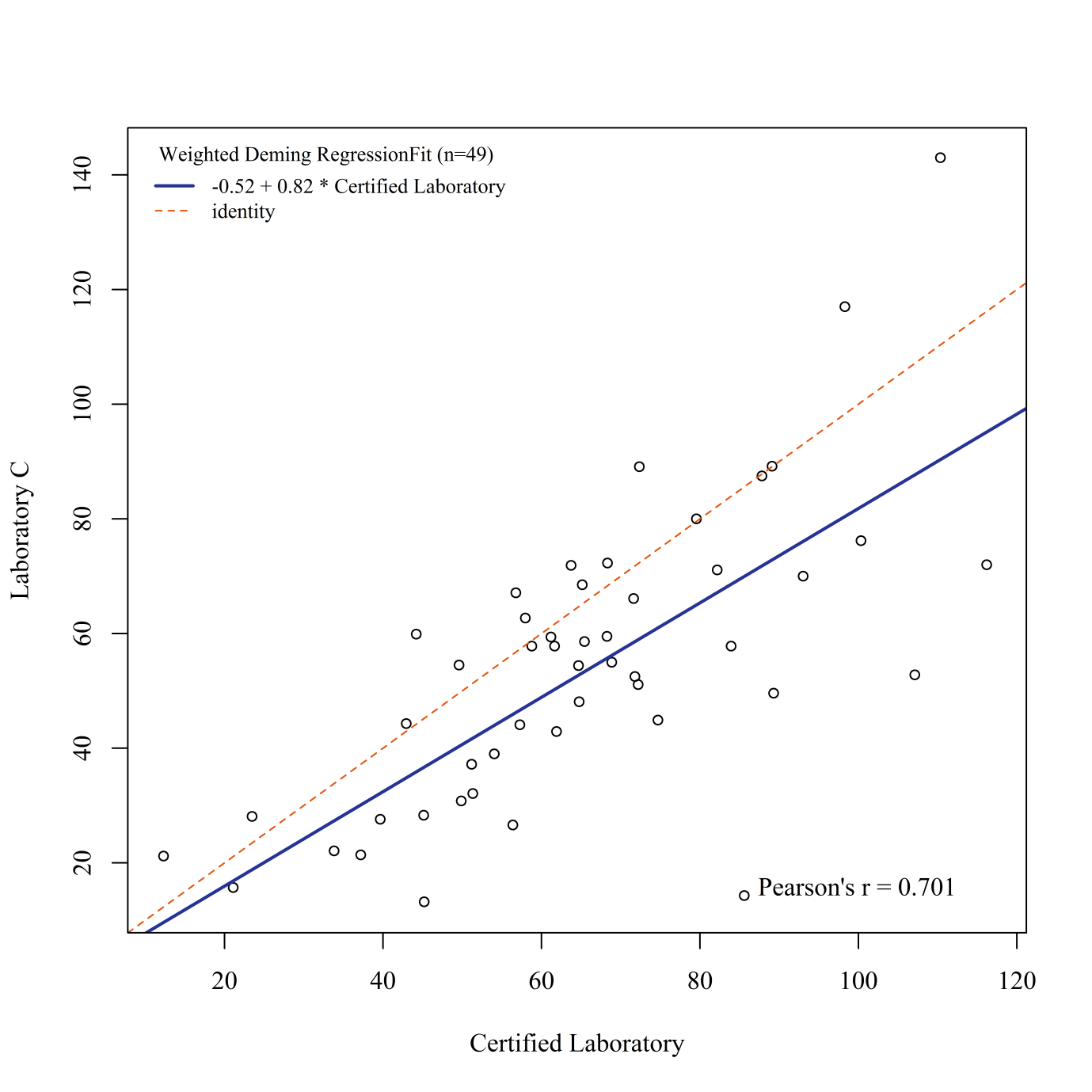


c)

Supplement: S1 Fig — Dotted lines show perfect agreement between assays (intercept of 0 and slope of 1). 25(OH)D, 25-hydroxyvitamin D; LC-MS/MS, liquid chromatography-tandem mass spectrometry (DOCX) [file pone.0135478.s001.docx]
